# Optimizing Hepatitis C Virus Antibody Testing Strategy and Setting: Results From a Large Real-World Screening Program

**DOI:** 10.1093/ofid/ofag218

**Published:** 2026-07-01

**Authors:** Grishma Hirode, Camelia Capraru, Aaron Vanderhoff, Andrew B Mendlowitz, Brett Wolfson-Stofko, Joel Karkada, David Smookler, Mediongo-Abasi Usoro, Steven M Friedman, Kathy Bates, Tony Mazzulli, Joshua V Juan, Hemant Shah, Bettina E Hansen, Harry L A Janssen, Mia J Biondi, Jordan J Feld

**Affiliations:** Toronto Centre for Liver Disease/Viral Hepatitis Care Network (VIRCAN), University Health Network, Toronto, Ontario, Canada; Toronto Centre for Liver Disease/Viral Hepatitis Care Network (VIRCAN), University Health Network, Toronto, Ontario, Canada; Toronto Centre for Liver Disease/Viral Hepatitis Care Network (VIRCAN), University Health Network, Toronto, Ontario, Canada; Toronto Centre for Liver Disease/Viral Hepatitis Care Network (VIRCAN), University Health Network, Toronto, Ontario, Canada; Institute of Health Policy, Management and Evaluation, University of Toronto, Toronto, Ontario, Canada; Toronto Centre for Liver Disease/Viral Hepatitis Care Network (VIRCAN), University Health Network, Toronto, Ontario, Canada; Center for Drug Use and HIV/HCV Research (CDUHR), School of Global Public Health, NewYork University, New York, New York, USA; Toronto Centre for Liver Disease/Viral Hepatitis Care Network (VIRCAN), University Health Network, Toronto, Ontario, Canada; Toronto Centre for Liver Disease/Viral Hepatitis Care Network (VIRCAN), University Health Network, Toronto, Ontario, Canada; Toronto Centre for Liver Disease/Viral Hepatitis Care Network (VIRCAN), University Health Network, Toronto, Ontario, Canada; Department of Emergency Medicine, University Health Network, Toronto, Ontario, Canada; Emergency Department, Toronto Western Hospital, University Health Network, Toronto, Ontario, Canada; Department of Laboratory Medicine and Pathobiology, University of Toronto, Toronto, Ontario, Canada; Department of Microbiology, University Health Network/Sinai Health System, Toronto, Ontario, Canada; Hepatology Division, Albany Medical Clinic, Toronto, Ontario, Canada; Toronto Centre for Liver Disease/Viral Hepatitis Care Network (VIRCAN), University Health Network, Toronto, Ontario, Canada; Academics, William Osler Health System, Brampton, Ontario, Canada; Department of Epidemiology and Biostatistics, Erasmus MC University Medical Center, Rotterdam, Netherlands; Department of Gastroenterology and Hepatology, Erasmus MC University Medical Center, Rotterdam, the Netherlands; Toronto Centre for Liver Disease/Viral Hepatitis Care Network (VIRCAN), University Health Network, Toronto, Ontario, Canada; School of Nursing, York University, Toronto, Ontario, Canada; Toronto Centre for Liver Disease/Viral Hepatitis Care Network (VIRCAN), University Health Network, Toronto, Ontario, Canada

**Keywords:** barriers, cascade, hepatitis C, prevalence, real-world

## Abstract

**Background:**

Optimal hepatitis C virus (HCV) screening is required to prevent transmission and progression to advanced liver disease and to meet global elimination targets. While administrative health data can highlight trends in testing and treatment, additional information is required to understand barriers in the care cascade. We conducted a real-world analysis documenting the care cascade and the effectiveness of HCV antibody (Ab) screening modalities across different community and clinical settings.

**Methods:**

This was a cross-sectional study of individuals who completed HCV Ab testing at participating centers in Ontario, Canada, between May 2010 and June 2023.

**Results:**

Among 61 605 individuals tested, the prevalence of HCV Ab was 13.6% and highest among individuals aged 35–44 years (23.2%) and individuals tested at addiction clinics (41.0%). Among those with an HCV Ab–positive result, 79.7% ever completed RNA testing of whom 57.3% were RNA positive. Among those with an RNA-positive result, 65.0% attended a follow-up appointment. Among those who attended their appointment, 94.0% initiated treatment (61.1% of the RNA-positive), 77.4% of those who started had documented treatment completion (47.3% of the RNA-positive), and 59.6% of those who completed treatment had documented sustained virologic response 12 weeks after treatment (28.2% of the RNA-positive). The Ab testing modality was closely related to test setting, and primary care was the most effective at linkage to care.

**Conclusions:**

While there was some attrition from HCV Ab positivity to RNA testing, treatment initiation was higher compared with administrative data. These results highlight the benefits of multiple testing modalities and matching the optimal Ab testing modality to the setting, with strong pathways for linkage to care.

Because hepatitis C virus (HCV) infection constitutes a major public health threat in Canada, Canada has committed to the global strategy to achieve HCV elimination by 2030 [1, 2]. Left untreated, HCV can lead to cirrhosis and hepatocellular carcinoma, contributing to significant morbidity and mortality rates. The World Health Organization estimated that 50 million persons are living with HCV infection globally [2]. Based on surveillance data, in Canada, the prevalence has been estimated to be 30.4 per 100 000 people, and based on national data, there is a higher prevalence among males and individuals aged 25–39 years [3].

With improved testing methods and widespread availability of highly effective direct-anting antivirals, optimal screening and linkage to care is crucial to meet elimination targets. Currently in Canada, the Canadian Association for the Study of the Liver [4] and the Canadian Task Force for Preventive Health Care [5] have differing screening recommendations. While both agree on risk-based screening to identify undiagnosed HCV and new infections, the Canadian Task Force for Preventive Health Care recommends only risk-based screening, whereas the Canadian Association for the Study of the Liver also recommends one-time screening for all individuals born from 1945 to 1975. However, both were published before the United States adopted universal screening of all adults [6]. Existing studies using administrative datasets or involving individuals at elevated risk have identified some of the barriers to successful linkage to care and treatment uptake [7–12].

These barriers typically include patient-level factors, such as poverty, mental health and substance use disorders, as well as system- or provider-level factors, including limited primary care capacity to initiate treatment, geographic inequities in access to specialist services, stigma that can deter engagement, administrative delays, low health literacy, and linguistic and cultural barriers. These barriers disproportionately affect priority populations, such as people who inject drugs and newcomers to Canada, contributing to persistent attrition across the HCV care continuum despite the availability of highly effective direct-anting antivirals. These challenges are further compounded by poor service integration, with separate systems for harm reduction, primary care, and specialist treatment creating obstacles to coordinated, “one-stop-shop” HCV care models.

The main aim of the current study was to document the clinical cascade of care using data from the implementation of HCV antibody (Ab) screening. This study used real-world data captured from a large cohort of individuals who were screened for HCV Ab using multiple Ab testing modalities across various clinical and community settings. This undertaking was part of targeted efforts led by the Viral Hepatitis Care Network, or VIRCAN, which partners with a range of different organizations across Canada, mostly in Ontario, to provide HCV education, promote research, and develop novel models of care to achieve elimination. While point-of-care testing (POCT) may facilitate widespread screening, these tests are generally underused, and the impact of using POCT on the cascade of care is not well described [13–15]. This is especially true when considering settings that provide care to diverse underserved communities. Therefore, the secondary aim was to determine the impact and utility of screening by Ab testing modality, test setting, and the patient population.

## METHODS

HCV Ab screening was conducted in multiple settings across Ontario, Canada, between May 2010 and June 2023, with the vast majority of testing conducted after 2015 (99.9%). HCV Ab testing was performed by means of conventional phlebotomy-based testing, the OraQuick HCV rapid Ab POCT, or a dried-blood spot (DBS) collection card. Follow-up HCV RNA testing and linkage to care after a positive Ab test result was site specific. However, typically, positive POCT Ab results led to DBS collection for HCV RNA testing, positive DBS Ab results led to reflex HCV RNA testing from the same sample, and positive Ab results obtained with phlebotomy-based testing required a second sample collection for HCV RNA testing. Reflex testing was not available for phlebotomy-based Ab testing in Ontario when this study was conducted. The turnaround time for phlebotomy-based and DBS testing was 1–2 weeks for both Ab and RNA results, and the turnaround time for POCT was 20 minutes for Ab results. Deidentified data were entered into a standardized case report form by each center and sent to the central site at the Toronto Centre for Liver Disease (University Health Network, Canada). The collection of these data has received a research ethics board waiver from the University Health Network (no. 17-0136).

### Study Population and Variables

Individuals who completed HCV Ab testing at a participating center or community setting were included. Centers were categorized based on setting and patient population served, as follows: primary care or human immunodeficiency virus (HIV) preexposure prophylaxis (PrEP) clinic, emergency department or walk-in clinic, screening event, community outreach, addiction clinic, hepatology clinic, or pharmacy [16]. Primary care included family health teams, community health centers, nurse practitioner–led clinics, and solo/team family medicine physician practices. Screening events were defined as one-time events such as health fairs and screening events held in hospital lobbies. Community outreach was characterized as screening events for higher-risk individuals in organizations, such as shelters or drop-in centers, offered on multiple occasions at a certain frequency. Addiction clinics included opioid agonist treatment clinics, rapid access addiction medicine clinics, and drug recovery and rehabilitation centers.

Demographic data included age at the time of screening and gender (including self-reported gender, record-reported gender, or biological sex). Screening data included the date of the HCV Ab test, Ab status, type of Ab test, and subsequent results in the cascade of care, including RNA testing completion after a positive Ab test result, RNA test results, attendance at the first appointment after a positive RNA test result, documented treatment initiation, documented treatment completion, through documented sustained virologic response 12 weeks after treatment (SVR12). The HCV Ab status was categorized as “known positive” if the individual was aware of a previous positive test result, had previously started treatment, or based on available historical data, and as “new positive” if the individual was unaware of their testing history or results, or if previous testing data was unavailable. The primary outcome was the prevalence HCV Ab positivity and subsequent linkage to care for HCV RNA–positive individuals by setting.

### Statistical Analysis

Participant characteristics are presented as numbers and percentages for binary and categorical variables and as mean and SD for continuous variables. We examined differences in HCV Ab prevalence and engagement in the care cascade by participant characteristics. Multivariable logistic regression models were used to evaluate factors (age at the time of screening, gender, Ab status, type of Ab test, and test setting) associated with progression through specific points early in the cascade of care (completion of RNA testing after a positive Ab test result, attendance at the first appointment after a positive RNA test result, and documented treatment initiation). When examining differences by type of Ab test, the “historical test result” category was excluded. Hepatology clinics and pharmacies included HCV testing of individuals for other types of care and referrals for other liver diseases. Thus, hepatology clinics and pharmacies were excluded from the analyses when we evaluated differences by type of Ab test setting. Between-group comparisons used χ^2^ tests, Student *t* tests, or 1-way analysis of variance, as appropriate. Two-tailed *P* values <.05 were considered statistically significant. Statistical analyses used STATA software, version 15.0 (StataCorp).

## RESULTS

**Figure 1.**
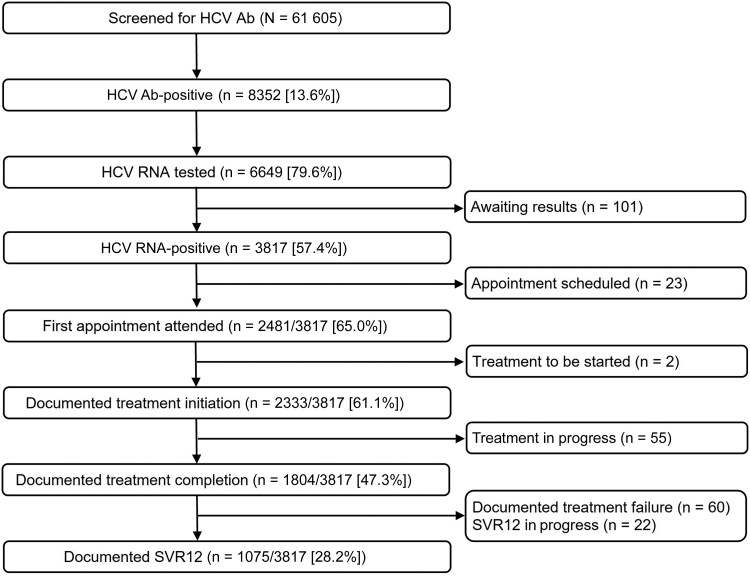
Flowchart depicting the overall cascade of care. Abbreviations: Ab, antibody; HCV, hepatitis C virus; SVR12, sustained virologic response 12 weeks after treatment.

Of 61 605 HCV Ab tests conducted, 13.6% (n = 8352) had positive results, of which 50.2% were known HCV Ab-positive (n = 4194) and 49.7% newly identified HCV Ab–positive (n = 4151) individuals (Figure 1 and Table 1). Among the total cohort, the mean age (SD) at the time of testing was 47.3 (14.9) years, and 51.1% of those tested were women (n = 31 482). Most Ab testing used phlebotomy-based testing (49.9%) or POCT (41.5%), and 38.8% of testing occurred in primary care settings (n = 23 879) (Table 1).

**Table 1.** Demographic Characteristics of Screened Participants: Total and by Hepatitis C Virus Antibody–Positive Status

Characteristic, No. (%)^a^		
	Total (N = 61 605)	HCV Ab-Positive (n = 8352 [13.6])
HCV Ab-positive status		
Known	4194 (6.8)	4194 (50.2)
New	4151 (6.7)	4151 (49.7)
Type of Ab test		
Historical test result	1556 (2.5)	1559 (18.7)
Phlebotomy	30 754 (49.9)	2326 (27.9)
POCT	25 586 (41.5)	3173 (38.0)
DBS	3244 (5.3)	839 (10.1)
Type of Ab test setting		
Primary care/PrEP clinic	23 879 (38.8)	383 (4.6)
ED/walk-in clinic	3946 (6.4)	58 (0.7)
Screening event	6887 (11.2)	266 (3.2)
Community outreach	12 234 (19.9)	4118 (49.3)
Addiction clinic	7658 (12.4)	3138 (37.6)
Hepatology clinic	5475 (8.9)	17 (0.2)
Pharmacy	1526 (2.5)	371 (4.6)
Age at screening, mean (SD), y^b^	47.3 (14.9)	43.5 (11.5)
Age at screening		
<25 y	2935 (4.8)	197 (2.4)
25–34 y	10 209 (16.6)	1863 (22.3)
35–44 y	11 629 (18.9)	2701 (32.3)
45–54 y	11 756 (19.1)	1863 (22.3)
55–64 y	11 110 (18.0)	1280 (15.3)
≥65 y	7834 (12.7)	355 (4.3)
Gender^c^		
Woman	31 482 (51.1)	2987 (35.8)
Man	29 852 (48.5)	5296 (63.4)
Other	139 (0.2)	40 (0.5)

Abbreviations: Ab, antibody; DBS, dried-blood spot; ED, emergency department; HCV, hepatitis C virus; POCT, point-of-care testing; PrEP, preexposure prophylaxis.

^a^Data represent no. (%) of participants unless otherwise specified.

^b^Age at screening was missing for 10.0% of the cohort.

^c^Gender was missing for 0.2% of the cohort.

Characteristics of individuals with an HCV Ab–positive result are displayed in Table 1. Individuals aged 35–44 years had the highest HCV Ab–positive prevalence (23.2%), followed by those aged 25–34 years (18.3%) (*P* < .001 comparing prevalence across all age groups), and the prevalence was higher among men (17.7%) than among women (9.5%) (*P* < .001). When stratified by both age and gender, HCV Ab–positive prevalence varied slightly by age group across gender. However, men and women in the age range of 35–44 years had the highest proportions of HCV Ab positivity (Supplementary Figure 1).

The HCV Ab status of the tested individuals, Ab testing modality, and Ab test setting were closely related (Supplementary Tables 1 and 2). The highest HCV Ab–positive prevalence was at addiction clinics (41.0%), followed by community outreach events (33.7%) (*P* < .001). Individuals tested at these settings were generally younger and had a higher proportion of men compared with other test settings. These settings also had higher proportions of individuals with known HCV Ab–positive status and primarily used POCT for Ab testing (Supplementary Tables 1 and 2). Phlebotomy-based Ab testing was mostly used at primary care/PrEP clinics, and DBS testing was used at emergency departments or walk-in clinics.

### Cascade of Care

Among individuals with an HCV Ab–positive test result, 79.7% were tested for HCV RNA, and 57.3% of those tested were HCV RNA-positive (Figure 1). Among those with an HCV RNA–positive test result, 65.0% attended the follow-up appointment. While there was some loss to follow-up after the first appointment, the treatment initiation rate thereafter was excellent, with 94.0% of those who attended their appointment starting treatment (61.1% of the RNA-positive), and 77.4% of those who started the treatment regimen having documented treatment completion (47.3% of the RNA-positive). Among those with documented treatment completion, 59.6% had documented SVR12 (28.2% of the RNA-positive). Among patients who completed SVR12 testing, 3.3% (n = 60) had a detectable viral load.

### HCV Ab Positive Status

**Figure 2.**
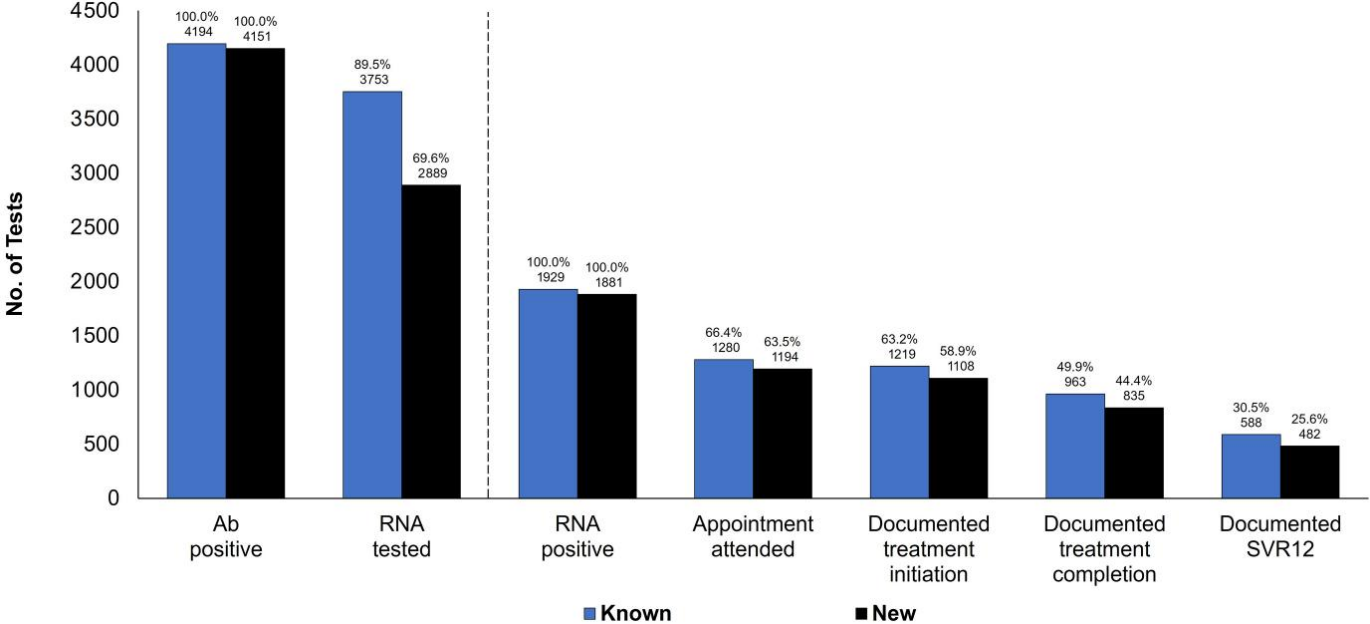
Cascade of care by hepatitis C virus antibody (Ab) positivity status. Abbreviation: SVR12, sustained virologic response 12 weeks after treatment.

On adjusted multivariable regression, newly identified Ab-positive individuals were less engaged early in the cascade than those with known Ab-positive status, and they had significantly lower odds of completing an RNA test (69.6% vs 89.5%; odds ratio [OR], 0.74 [95% confidence interval (CI), .63–.88]) and attending their first appointment after an RNA-positive test result (63.5% vs 66.4%; OR, 0.71 [95% CI, .59–.85]) (Figure 2 and Table 2). Compared with newly identified Ab-positive individuals, those with known Ab-positive status also had better engagement further down the cascade of care, with a higher proportion of individuals starting treatment among those who attended their first appointment (95.2% vs 92.8%), a higher proportion of documented treatment completion among those who started treatment (79.1% vs 75.4%), and a higher proportion of documented SVR12 among those who completed treatment (61.1% vs 57.7%).

**Table 2.** Multivariable Logistic Regression Models for Factors Associated With Progression Early in the Cascade of Care

Factor	OR (95% CI)		
	Completed RNA Testing After an Ab-Positive Result (n = 5035)	Attended 1st Appointment After an RNA-Positive Result (n = 2802)	Had Documented Treatment Initiation After Attending the 1st Appointment (n = 1839)
HCV Ab–positive status			
Known	1.00 (Reference)	1.00 (Reference)	1.00 (Reference)
New	0.77 (.64–.91)^a^	0.71 (.59–.85)^a^	0.66 (.31–1.45)
Type of Ab test			
Phlebotomy	1.00 (Reference)	1.00 (Reference)	1.00 (Reference)
POCT	0.20 (.16–.23)^a^	1.12 (.92–1.36)	0.68 (.36–1.29)
DBS	…	0.43 (.34–.55)^a^	0.52 (.14–1.93)
Ab test setting			
Primary care/PrEP clinic	1.00 (Reference)	1.00 (Reference)	1.00 (Reference)
ED/walk-in clinic	9.33 (1.12–78.0)^a^	0.18 (.08–.43)^a^	0.47 (.08–2.83)
Screening event	2.49 (1.56–3.97)^a^	0.19 (.09–.37)^a^	0.39 (.12–1.32)
Community outreach	2.23 (1.61–3.10)^a^	0.47 (.30–.73)^a^	15.2 (5.49–42.2)^a^
Addiction clinic	1.90 (1.34–2.68)^a^	0.44 (.27–.71)^a^	0.85 (.36–2.03)
Age at screening (y)	1.00 (.99–1.01)	1.02 (1.01–1.02)^a^	1.01 (.99–1.03)
Gender			
Woman	1.00 (Reference)	1.00 (Reference)	1.00 (Reference)
Man	1.09 (.95–1.25)	0.95 (.80–1.13)	1.02 (.61–1.71)

Abbreviations: Ab, antibody; CI, confidence interval; DBS, dried-blood spot; ED, emergency department; HCV, hepatitis C virus; OR, odds ratio; POCT, point-of-care testing; PrEP, preexposure prophylaxis.

^a^Statistically significant at *P* < .05.

### HCV Ab Testing Modality

**Figure 3.**
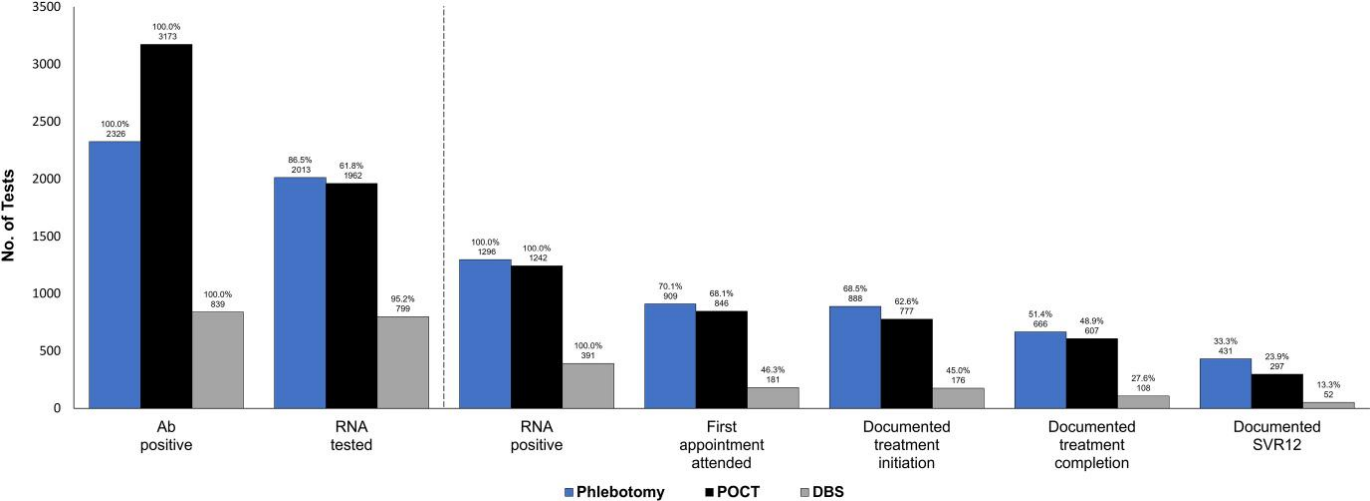
Cascade of care by hepatitis C virus antibody (Ab) testing modality. Abbreviations: DBS, dried blood spot; POCT, point-of-care testing; SVR12, sustained virologic response 12 weeks after treatment.

On adjusted multivariable regression, because DBS testing involves reflex RNA testing, compared with phlebotomy-based testing, DBS testing had significantly higher odds of completion of RNA testing after an HCV Ab–positive test result (95.2% vs 86.5%; OR, 1.89 [95% CI, 1.32–2.70]) but lower odds of attendence at the first appointment after an RNA-positive test result (46.3% vs 70.1%; OR, 0.43 [95% CI, .34–.55]) (Figure 3 and Table 2). Compared with phlebotomy-based testing, POCT had significantly lower odds of RNA test completion (61.8%; OR, 0.20 [95% CI, .16–.24]). After attendance at the first appointment, the testing modalities showed similar engagement later in the cascade of cade. The proportion of individuals who started treatment among those who attended their first appointment was >90% for all 3 modalities. Among those who started treatment, phlebotomy-based testing (75.0%) and POCT (78.1%) had higher proportions of documented treatment completion compared with DBS (61.4%) and a higher proportion of documented SVR12 among those who completed treatment (phlebotomy, 64.7%; POCT, 48.9%; DBS, 48.1%).

### HCV Ab Test Setting

**Figure 4.**
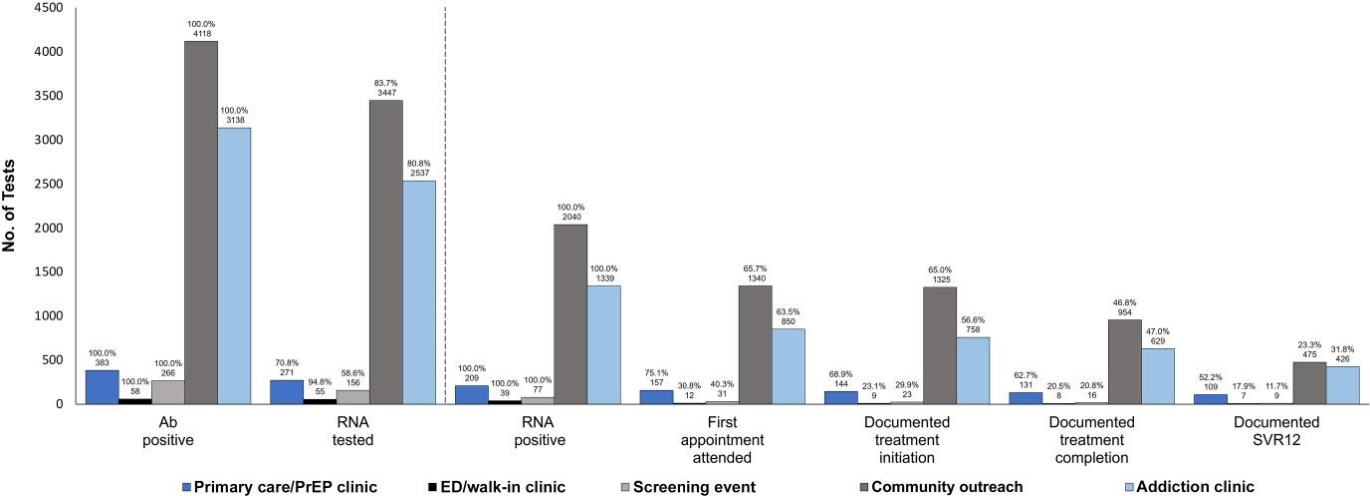
Cascade of care by hepatitis C virus test setting. Abbreviations: Ab, antibody; ED, emergency department; PrEP, preexposure prophylaxis; SVR12, sustained virologic response 12 weeks after treatment.

On adjusted multivariable regression, all test settings had significantly higher odds of completing RNA testing, compared with primary care/PrEP clinics. This is because the main testing modality at primary care/PrEP clinics was phlebotomy, which required additional steps. However, the odds of attendance at the first appointment after an RNA-positive test result was significantly lower at all settings (Table 2). Among patients who tested HCV RNA positive, primary care/PrEP clinics (75.1%), community outreach programs (65.7%), and addiction clinics (63.5%) had the highest attendance at the follow-up appointment, had higher proportions of individuals who started treatment, and higher rates of documented treatment completion (Figure 4). While community outreach programs showed significantly higher odds of starting treatment compared with primary care/PrEP clinics among RNA-positive individuals who attended their first appointment (98.9% vs 91.7%; OR, 15.2 [95% CI, 5.49–42.2]), individuals tested at primary care/PrEP clinics were relatively more engaged throughout the cascade of care. Patient engagement throughout the cascade of care was generally lower for screening events, with particularly low SVR12 documentation rates (11.7% of the RNA positive).

## DISCUSSION

This large, real-world study demonstrates the differences in HCV Ab–positive prevalence based on age, gender, Ab testing modality, and test setting, signifying that implementation of setting-specific screening strategies may be necessary, particularly for the diagnosis of new infections and subsequent linkage to care. It also identifies gaps in the clinical cascade of care, including nuances not captured in administrative data, such as appointment attendance and treatment completion, which may affect cure rates.

Similar to results from Canadian surveillance data [3], men and individuals aged 25–44 years had the highest HCV Ab prevalence in this study. These data support screening strategies that more comprehensively include younger individuals in cohort screening approaches. With respect to test setting, community outreach and addiction clinics had the highest prevalence. The results of this study are in line with higher rates of injection drug use among young adults, particularly among young men, highlighting the need to expand access to harm reduction services and HCV education among this population [11, 12, 17–21]. Furthermore, stigma within the healthcare sector toward people who inject drugs may further complicate screening and treatment efforts among this population and needs to be systemically addressed [10, 22–26]. However, addiction clinics often provide continuity of care and therefore are ideal for higher engagement and better progression through the cascade [27].

In this study, primary care, PrEP clinics, and addiction clinics were the most successful settings for effective linkage to care of RNA-positive individuals. Notably, people screened in these settings who tested positive were given an appointment for follow-up at the time of the positive test result. These differences in linkage to care by test setting reflect differences in the populations served and available resources. Given that the Ab testing modality was closely related to the setting, matching the optimal Ab screening modality to the test setting will be critical to reach elimination targets. For example, while phlebotomy-based testing in primary care was most successful for linkage to care, this is likely more reflective of the population being tested than the strategy. People attending appointments in primary care settings are likely more engaged in their health needs and thus more likely to follow up than those approached with POCT at an outreach event. However, POCT facilitates rapid diagnosis and is very useful in high throughput and low-barrier settings.

Testing modalities have evolved over time with increasing access to POCT and DBS. Given the relatively high completion rates with POCT, it will be the key screening approach in expanding community outreach. In the current study, many test settings used POCT, indicating that POCT may be a feasible alternative to conventional screening methods. High rates of RNA testing using DBS at emergency departments/walk-in clinics highlight the value of reflex RNA testing. At present, however, DBS is not widely available. The ability to follow POCT Ab testing with POCT RNA testing would likely be a preferred strategy in many settings, although DBS RNA follow-up is also a reasonable choice and facilitated linkage in our study despite the slower turnaround time for RNA results. Nevertheless, compared with DBS, immediate HCV Ab–positive test results from POCT may improve linkage even if RNA status is unknown because the patient does not receive their DBS Ab result for days to weeks, even though both Ab and RNA are completed from the same DBS collection card.

Overall, there were 2 major drops in the cascade of care. The first was in the scheduling of an appointment after an HCV RNA–positive test result, whereby only 65.0% of HCV RNA–positive participants attended a follow-up appointment. The second major drop was after treatment completion for SVR12 testing across most settings. A proposed strategy to improve linkage to care would be to schedule a follow-up appointment at the time of RNA testing, which can later be cancelled in case of a negative test result. Previous studies have shown that using electronic medical/health record reminders and alerts have enhanced HCV testing rates [28–31]. Similarly, an SVR12 testing appointment could be set at the time of treatment initiation, with appointment reminders, to improve SVR12 testing completion rates. However, the actual need for SVR12 testing among individuals without advanced liver disease or extrahepatic manifestations, particularly those without active risk factors for reinfection, is becoming less clear given the near-perfect cure rates of current therapeutic agents, provided adherence to the treatment regimen; even patients not tested for SVR12 likely achieved SVR12 [2, 32, 33].

We also observed a high proportion of older participants among those with a HCV Ab–positive result, particularly those belonging to the baby boomer birth cohort (born in 1945–1975) [4, 16]. The HCV Ab prevalence among baby boomers in Canada remains high while testing rates remain low. Although prioritizing testing among baby boomers may be useful, it is important to recognize that this population is aging and has many competing healthcare risks [34–38]. British Columbia has recently adopted birth cohort screening, whereas other provinces continue to rely on risk-based screening. Universal screening may be cost-effective based on published data from other global regions; however, studies specific to Canada would be helpful to account for differences in the health system and related costs [39–41].

The current study has limitations. First, it used cross-sectional testing data based on HCV Ab testing. As a result, an individual may have been included in the cohort more than once if they were tested across different sites or at different time points. However, once Ab tested, that individual would have been followed through the HCV care cascade by the testing site. Given the very large study population, this is unlikely to have affected the results significantly. Second, this study used convenience sampling and includes data solely from partner organizations. Thus, the data may be biased toward specific test settings, and engagement in the cascade might be underestimated if a participant continued care with a non-partner organization. Third, there may be misclassification bias, and the results need to be interpreted in the context of this study. For example, an individual with a previous positive HCV Ab result, who may have even received that result, may be misclassified as a newly identified Ab-positive individual due to unawareness of HCV status and the absence of accessible historical test result at that testing site. Therefore, while this study shows that certain test settings would be more effective, the generalizability and cost-effectiveness of universal screening based on these findings warrant further research. In addition, since this study was completed, Ontario has implemented reflex testing on blood samples. Thus, gaps in care from screening to diagnosis observed may be less relevant in the future.

In conclusion, our data highlight specific strategies to enhance patient engagement and follow-up and provide more detailed data on the steps involved in the HCV care cascade, an important aspect not typically captured in administrative health data. Our results demonstrate that identifying the most effective setting-specific testing modality based on the patient population, staff, and available resources should be evaluated during the development of an HCV screening program.

## Supplementary Material

ofag218_Supplementary_Data

## Supplementary Data

Supplementary materials are available at *Open Forum Infectious Diseases* online. Consisting of data provided by the authors to benefit the reader, the posted materials are not copyedited and are the sole responsibility of the authors, so questions or comments should be addressed to the corresponding author.

## References

[1] Kwong JC, Ratnasingham S, Campitelli MA, et al The impact of infection on population health: results of the Ontario burden of infectious diseases study. PLoS One 2012; 7:e44103. 22962601 10.1371/journal.pone.0044103 PMC3433488

[2] World Health Organization . Global Hepatitis Report 2017. Geneva: World Health Organization; 2017. Available at: https://iris.who.int/handle/10665/255016.

[3] Public Health Agency of Canada . Hepatitis C in Canada: 2019 surveillance data. Public Health Agency of Canada; 2019. Available at: https://www.canada.ca/en/public-health/services/publications/diseases-conditions/hepatitis-c-2019-surveillance-data.html.

[4] Shah H, Bilodeau M, Burak KW, et al The management of chronic hepatitis C: 2018 guideline update from the Canadian Association for the Study of the Liver. Can Med Assoc J 2018; 190:E677–87. 29866893 10.1503/cmaj.170453 PMC5988519

[5] Grad R, Thombs BD, Tonelli M, et al Recommendations on hepatitis C screening for adults. Can Med Assoc J 2017; 189:E594–604. 28438952 10.1503/cmaj.161521 PMC5403642

[6] Schillie S, Wester C, Osborne M, Wesolowski L, Ryerson AB. CDC recommendations for hepatitis C screening among adults—United States, 2020. Morb Mortal Wkly Rep 2020; 69:1–17. 10.15585/mmwr.rr6902a1 PMC7147910 32271723

[7] Yasseen AS, Kwong JC, Feld JJ, et al Viral hepatitis C cascade of care: a population-level comparison of immigrant and long-term residents. Liver Int 2021; 41:1775–88. 33655665 10.1111/liv.14840

[8] Cowan E, Hardardt J, Brandspiegel S, Eiting E, Calderon Y. Care cascade of patients with hepatitis C and HIV identified by emergency department screening. J Viral Hepat 2021; 28:1484–7. 33932240 10.1111/jvh.13529

[9] Greenaway C, Azoulay L, Allard R, et al A population-based study of chronic hepatitis C in immigrants and non-immigrants in Quebec, Canada. BMC Infect Dis 2017; 17:140. 28193199 10.1186/s12879-017-2242-y PMC5307836

[10] Wakeman SE, Rich JD. Barriers to post-acute care for patients on opioid agonist therapy; an example of systematic stigmatization of addiction. J Gen Intern Med 2017; 32:17–9. 27393486 10.1007/s11606-016-3799-7 PMC5215148

[11] Grebely J, Raffa JD, Lai C, et al Low uptake of treatment for hepatitis C virus infection in a large community-based study of inner city residents. J Viral Hepat 2009; 16:352–8. 19226330 10.1111/j.1365-2893.2009.01080.x

[12] Canadian Network on Hepatitis C Blueprint Writing Committee and Working Groups . Blueprint to inform hepatitis C elimination efforts in Canada. **2019**. Available at: https://www.canhepc.ca/en/blueprint. Accessed 26 Apr 2026.

[13] Chevaliez S, Pawlotsky JM. New virological tools for screening, diagnosis and monitoring of hepatitis B and C in resource-limited settings. J Hepatol 2018; 69:916–26. 29800630 10.1016/j.jhep.2018.05.017

[14] Koo V, Tian F, Wong WWL. Cost-effectiveness analysis of hepatitis C virus (HCV) point-of-care assay for HCV screening. Liver Int 2022; 42:787–95. 34847288 10.1111/liv.15123

[15] Williams B, Howell J, Doyle J, et al Point-of-care hepatitis C testing from needle and syringe programs: an Australian feasibility study. Int J Drug Policy 2019; 72:91–8. 31129023 10.1016/j.drugpo.2019.05.012

[16] Biondi MJ, Hirode G, Capraru C, et al Birth cohort hepatitis C antibody prevalence in real-world screening settings in Ontario. Can Liver J 2022; 5:362–71. 36133900 10.3138/canlivj-2021-0036 PMC9473558

[17] Jacka B, Applegate T, Poon AF, et al Transmission of hepatitis C virus infection among younger and older people who inject drugs in Vancouver, Canada. J Hepatol 2016; 64:1247–55. 26924451 10.1016/j.jhep.2016.02.031 PMC4874854

[18] Centers for Disease Control and Prevention . Use of enhanced surveillance for hepatitis C virus infection to detect a cluster among young injection-drug users—New York, November 2004-April 2007. Morb Mortal Wkly Rep 2008; 57:517–21. 18480744

[19] Centers for Disease Control and Prevention . Hepatitis C virus infection among adolescents and young adults: Massachusetts, 2002–2009. Morb Mortal Wkly Rep 2011; 60:537–41. 21544042

[20] Centers for Disease Control and Prevention . Notes from the field: hepatitis C virus infections among young adults–rural Wisconsin, 2010. Morb Mortal Wkly Rep 2012; 61:358. 22592276

[21] Barocas JA, Brennan MB, Hull SJ, Stokes S, Fangman JJ, Westergaard RP. Barriers and facilitators of hepatitis C screening among people who inject drugs: a multi-city, mixed-methods study. Harm Reduct J 2014; 11:1–8. 24422784 10.1186/1477-7517-11-1 PMC3896714

[22] Merrill JO, Rhodes LA, Deyo RA, Marlatt GA, Bradley KA. Mutual mistrust in the medical care of drug users: the keys to the “narc” cabinet. J Gen Intern Med 2002; 17:327–33. 12047728 10.1046/j.1525-1497.2002.10625.x PMC1495051

[23] Elliott L, Bennett AS, Wolfson-Stofko B. Life after opioid-involved overdose: survivor narratives and their implications for ER/ED interventions. Addiction 2019; 114:1379–86. 30851220 10.1111/add.14608 PMC6626567

[24] Henderson S, Stacey CL, Dohan D. Social stigma and the dilemmas of providing care to substance users in a safety-net emergency department. J Health Care Poor Underserved 2008; 19:1336–49. 19029756 10.1353/hpu.0.0088

[25] Brener L, Von Hippel W, Kippax S, Preacher KJ. The role of physician and nurse attitudes in the health care of injecting drug users. Subst Use Misuse 2010; 45:1007–18. 20441447 10.3109/10826081003659543

[26] Van Boekel LC, Brouwers EPM, Van Weeghel J, Garretsen HFL. Stigma among health professionals towards patients with substance use disorders and its consequences for healthcare delivery: systematic review. Drug Alcohol Depend 2013; 131:23–35. 23490450 10.1016/j.drugalcdep.2013.02.018

[27] Handford C, Kahan M, Srivastava A, et al Buprenorphine/naloxone for opioid dependence: clinical practice guideline. Toronto: Centre for Addiction and Mental Health; 2011.

[28] Tapp H, Ludden T, Shade L, Thomas J, Mohanan S, Leonard M. Electronic medical record alert activation increase hepatitis C and HIV screening rates in primary care practices within a large healthcare system. Prev Med Reports 2020; 17:101036. 10.1016/j.pmedr.2019.101036 PMC6965743 31970042

[29] Al-hihi E, Shankweiler C, Stricklen D, Gibson C, Dunn W. Electronic medical record alert improves HCV testing for baby boomers in primary care setting: adults born during 1945–1965. BMJ Open Qual 2017; 6:e000084. 10.1136/bmjoq-2017-000084 PMC5704105 29209663

[30] Geboy AG, Nichols WL, Fernandez SJ, Desale S, Basch P, Fishbein DA. Leveraging the electronic health record to eliminate hepatitis C: screening in a large integrated healthcare system. PLoS One 2019; 14:e0216459. 31120906 10.1371/journal.pone.0216459 PMC6532960

[31] Federman AD, Kil N, Kannry J, et al An electronic health record–based intervention to promote hepatitis C virus testing among adults born between 1945 and 1965. Med Care 2017; 55:590–7. 28288075 10.1097/MLR.0000000000000715 PMC5759753

[32] Hashim A, Almahdi F, Albaba EA, et al Efficacy of DAAs in the treatment of chronic HCV: real-world data from the private health-care sector of the Kingdom of Saudi Arabia. J Epidemiol Glob Health 2020; 10:178–83. 32538035 10.2991/jegh.k.200117.002 PMC7310777

[33] Yee J, Carson JM, Hajarizadeh B, et al High effectiveness of broad access direct-acting antiviral therapy for hepatitis C in an Australian real-world cohort: the REACH-C study. Hepatol Commun 2022; 6:496–512. 34729957 10.1002/hep4.1826 PMC8870316

[34] Bolotin S, Feld JJ, Garber G, Wong WWL, Guerra FM, Mazzulli T. Population-based estimate of hepatitis C virus prevalence in Ontario, Canada. PLoS One 2018; 13:e0191184. 29360823 10.1371/journal.pone.0191184 PMC5779675

[35] Myers RP, Liu M, Shaheen AA. The burden of hepatitis C virus infection is growing: a Canadian population-based study of hospitalizations from 1994 to 2004. Can J Gastroenterol 2008; 22:381–7. 18414713 10.1155/2008/173153 PMC2662896

[36] Rotermann M, Langlois K, Andonov A, Trubnikov M. Seroprevalence of hepatitis B and C virus infections: results from the 2007 to 2009 and 2009 to 2011 Canadian Health Measures Survey. Heal Rep 2013; 24:3–13. 24259199

[37] Myers RP, Krajden M, Bilodeau M, et al Burden of disease and cost of chronic hepatitis C infection in Canada. Can J Gastroenterol Hepatol 2014; 28:243–50. 24839620 10.1155/2014/317623 PMC4049256

[38] Schanzer DL, Paquette D, Lix LM. Historical trends and projected hospital admissions for chronic hepatitis C infection in Canada: a birth cohort analysis. CMAJ Open 2014; 2:E139–44. 10.9778/cmajo.20130087 PMC4183164 25295233

[39] Eckman MH, Ward JW, Sherman KE. Cost effectiveness of universal screening for hepatitis C virus infection in the era of direct-acting, pangenotypic treatment regimens. Clin Gastroenterol Hepatol 2019; 17:930–9.e9. 30201597 10.1016/j.cgh.2018.08.080

[40] Deuffic-Burban S, Huneau A, Verleene A, et al Assessing the cost-effectiveness of hepatitis C screening strategies in France. J Hepatol 2018; 69:785–92. 30227916 10.1016/j.jhep.2018.05.027

[41] Zhou H, Yan M, Che D, Wu B. Universal screening for HCV infection in China: an effectiveness and cost-effectiveness analysis. JHEP Rep Innov Hepatol 2024; 6:101000. 10.1016/j.jhepr.2024.101000 PMC10933547 38481389

