# Supplementary material for: Optimizing Hepatitis C Virus Antibody Testing Strategy and Setting: Results From a Large Real-World Screening Program
Source: Open Forum Infect Dis. 2026 Jul 1;13(7):ofag218. doi: 10.1093/ofid/ofag218 (PMC13321397; doi:10.1093/ofid/ofag218)
Supplement: ofag218_Supplementary_Data [file ofag218_supplementary_data.docx]

**Supplemental Table 1**. Characteristics of screened participants by HCV Ab testing modality.

| **Characteristic** | **Type of Ab test** | | |
| --- | --- | --- | --- |
|  | **Phlebotomy** | **POCT** | **DBS** |
| No. of tests, n (%) | 30,754 (49.9) | 25,586 (41.5) | 3,244 (5.3) |
| HCV Ab-positive, n (%) | 2,326 (7.6) | 3,173 (12.4) | 839 (25.9) |
| Known | 1,111 (3.6) | 648 (2.5) | 443 (13.7) |
| New | 1,215 (4.0) | 2,525 (9.9) | 396 (12.2) |
| Age at screening, *years*, n (%) |  |  |  |
| <25 | 1,472 (4.8) | 1,385 (5.4) | 10 (0.3) |
| 25-34 | 3,742 (12.2) | 5,751 (22.5) | 186 (5.7) |
| 35-44 | 4,397 (14.3) | 6,138 (24.0) | 448 (13.8) |
| 45-54 | 5,189 (16.9) | 5,221 (20.4) | 919 (28.3) |
| 55-64 | 5,543 (18.0) | 4,336 (17.0) | 956 (29.5) |
| ≥65 | 4,642 (15.1) | 2,420 (9.5) | 724 (22.3) |
| Gender, n (%) |  |  |  |
| Woman | 18,616 (60.5) | 10,732 (41.9) | 1,412 (43.5) |
| Man | 12,100 (39.3) | 14,659 (57.3) | 1,829 (56.4) |
| Other | 14 (0.1) | 108 (0.4) | 3 (0.1) |

Ab, Antibody; DBS, Dried blood spot; HCV, Hepatitis C virus; POCT, Point-of-care testing.

**Supplemental Table 2.** Characteristics of screened participants by type of Ab test setting.

| **Characteristic** | **Type of Ab test setting** | | | | |
| --- | --- | --- | --- | --- | --- |
|  | **Primary care /**  **PrEP clinic** | **ED /**  **walk-in clinic** | **Screening event** | **Community outreach** | **Addiction clinic** |
| No. of tests, n (%) | 23,879 (38.8) | 3,946 (6.4) | 6,887 (11.2) | 12,234 (19.9) | 7,658 (12.4) |
| HCV Ab-positive, n (%) | 383 (1.6) | 58 (1.5) | 266 (3.9) | 4,118 (33.7) | 3,138 (41.0) |
| Known | 122 (0.5) | 3 (0.1) | 102 (1.5) | 2,213 (18.1) | 1,743 (22.8) |
| New | 260 (1.1) | 55 (1.4) | 164 (2.4) | 1,905 (15.6) | 1,389 (18.1) |
| Type of Ab test, n (%) |  |  |  |  |  |
| Historical test result | 58 (0.2) | 3 (0.1) | 93 (1.4) | 91 (0.7) | 1,314 (17.2) |
| Phlebotomy | 23,054 (96.6) | 0 (0) | 0 (0) | 1,882 (15.4) | 181 (2.4) |
| POCT | 740 (3.1) | 1,512 (38.3) | 6,791 (98.6) | 9,458 (77.3) | 5,740 (75.0) |
| DBS | 0 (0) | 2,431 (61.6) | 0 (0) | 783 (6.4) | 29 (0.4) |
| Age at screening, *years*, n (%) |  |  |  |  |  |
| <25 | 1,186 (5.0) | 82 (2.1) | 473 (6.9) | 341 (2.8) | 464 (6.1) |
| 25-34 | 2,801 (11.7) | 220 (5.6) | 1,285 (18.7) | 2,405 (19.7) | 2,331 (30.4) |
| 35-44 | 2,907 (12.2) | 397 (10.1) | 1,024 (14.9) | 3,577 (29.2) | 2,356 (30.8) |
| 45-54 | 3,636 (15.2) | 1,084 (27.5) | 1,357 (19.7) | 2,840 (23.2) | 1,380 (18.0) |
| 55-64 | 4,070 (17.0) | 1,190 (30.2) | 1,379 (20.0) | 2,251 (18.4) | 802 (10.5) |
| ≥65 | 3,507 (14.7) | 967 (24.5) | 1,170 (17.0) | 809 (6.6) | 224 (2.9) |
| Gender, n (%) |  |  |  |  |  |
| Woman | 15,369 (64.4) | 2,058 (52.2) | 3,712 (53.9) | 3,963 (32.4) | 2,970 (38.8) |
| Man | 8,483 (35.5) | 1,888 (47.9) | 3,057 (44.4) | 8,178 (66.9) | 4,659 (60.8) |
| Other | 6 (0.03) | 0 (0) | 27 (0.4) | 89 (0.7) | 17 (0.2) |

Ab, Antibody; DBS, Dried blood spot; ED, Emergency department; HCV, Hepatitis C virus; POCT, Point-of-care testing.

All between-group comparisons were significant with p<0.05.


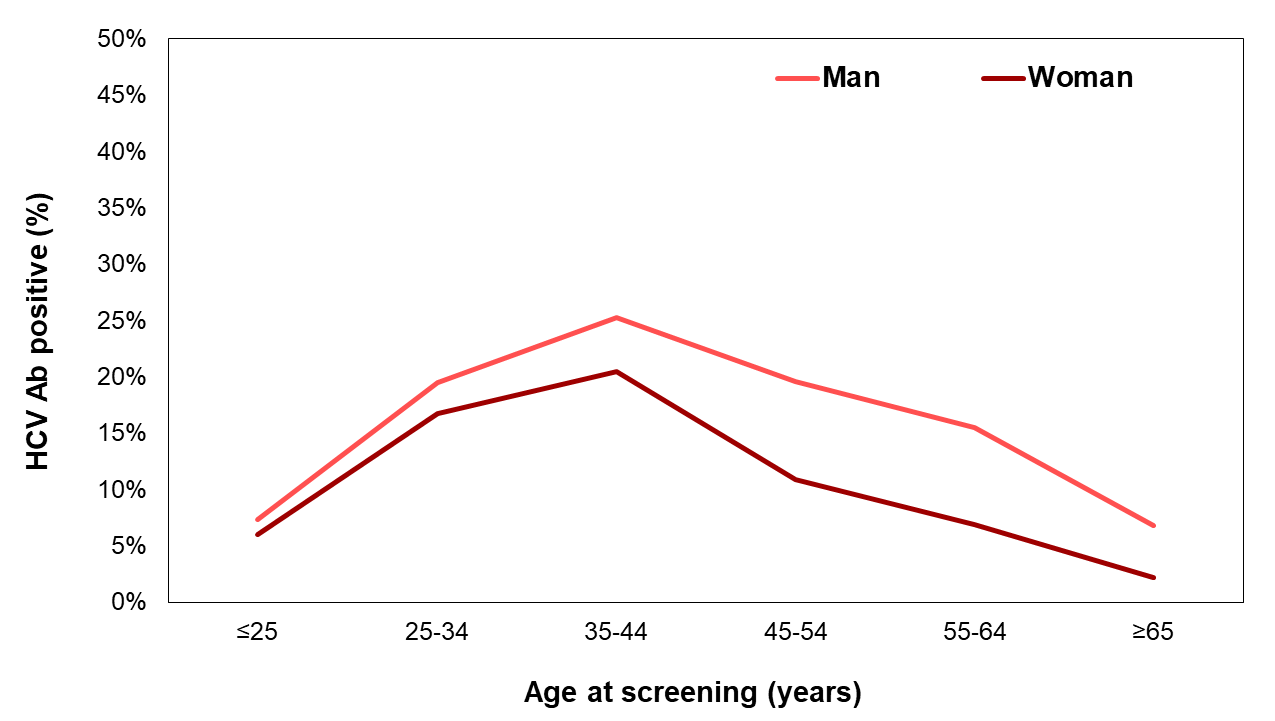


**Supplemental Figure 1.** HCV Ab positivity by age and gender.

Ab, Antibody; HCV, Hepatitis C virus.
